# Targeting the Formyl Peptide Receptor type 1 to prevent the adhesion of ovarian cancer cells onto mesothelium and subsequent invasion

**DOI:** 10.1186/s13046-019-1465-8

**Published:** 2019-11-08

**Authors:** Michele Minopoli, Giovanni Botti, Vincenzo Gigantino, Concetta Ragone, Sabrina Sarno, Maria Letizia Motti, Giosuè Scognamiglio, Stefano Greggi, Cono Scaffa, Maria Serena Roca, Maria Patrizia Stoppelli, Gennaro Ciliberto, Nunzia Simona Losito, Maria Vincenza Carriero

**Affiliations:** 1 Neoplastic Progression Unit, Istituto Nazionale Tumori IRCCS ‘Fondazione G. Pascale’, Via M.Semmola, 80131 Naples, Italy; 2 University of Campania “Luigi Vanvitelli”, Naples, Italy; 3 Pathology Unit, Istituto Nazionale Tumori IRCCS ‘Fondazione G. Pascale’, Naples, Italy; 4 University ‘Parthenope’, Naples, Italy; 5 Gynecologic Oncology, Istituto Nazionale Tumori IRCCS ‘Fondazione G. Pascale’, Naples, Italy; 6 Experimental Pharmacology Unit, Istituto Nazionale Tumori IRCCS ‘Fondazione G. Pascale’, Naples, Italy; 7 Institute of Genetics and Biophysics, National Research Council, Naples, Italy; 8 Istituto Nazionale Tumori IRCCS “Regina Elena”, Rome, Italy

**Keywords:** Formyl peptide receptor, Urokinase receptor, Peptide, Ovarian cancer, Adhesion, Mesothelium

## Abstract

**Background:**

The biological behavior of epithelial ovarian cancer (EOC) is unique since EOC cells metastasize early to the peritoneum. Thereby, new anti-target agents designed to block trans-coelomic dissemination of EOC cells may be useful as anti-metastatic drugs. The Urokinase Plasminogen Activator Receptor (uPAR) is overexpressed in EOC tissues, and its truncated forms released in sera and/or ascitic fluid are associated with poor prognosis and unfavorable clinical outcome. We documented that uPAR triggers intra-abdominal dissemination of EOC cells through the interaction of its 84–95 sequence with the Formyl Peptide Receptor type 1 (FPR1), even as short linear peptide Ser-Arg-Ser-Arg-Tyr (SRSRY). While the pro-metastatic role of uPAR is well documented, little information regarding the expression and role of FPR1 in EOC is currently available.

**Methods:**

Expression levels of uPAR and FPR1 in EOC cells and tissues were assessed by immunofluorescence, Western blot, or immunohystochemistry. Cell adhesion to extra-cellular matrix proteins and mesothelium as well as mesothelium invasion kinetics by EOC cells were monitored using the xCELLigence technology or assessed by measuring cell-associated fluorescence. Cell internalization of FPR1 was identified on multiple z-series by confocal microscopy. Data from in vitro assays were analysed by one-way ANOVA and post-hoc Dunnett t-test for multiple comparisons. Tissue microarray data were analyzed with the Pearson’s Chi-square (χ^2^) test.

**Results:**

Co-expression of uPAR and FPR1 by SKOV-3 and primary EOC cells confers a marked adhesion to vitronectin. The extent of cell adhesion decreases to basal level by pre-exposure to anti-uPAR84–95 Abs, or to the RI-3 peptide, blocking the uPAR84–95/FPR1 interaction. Furthermore, EOC cells exposed to RI-3 or desensitized with an excess of SRSRY, fail to adhere also to mesothelial cell monolayers, losing the ability to cross them. Finally, primary and metastatic EOC tissues express a high level of FPR1.

**Conclusions:**

Our findings identify for the first time FPR1 as a potential biomarker of aggressive EOC and suggests that inhibitors of the uPAR84–95/FPR1 crosstalk may be useful for the treatment of metastatic EOC.

## Background

Epithelial ovarian cancer (EOC) is the 8th most common cause of death from cancer in women worldwide and its incidence rate is predicted to increase by 55% by 2035 [1–3]. Nowadays, the treatment for EOC is aggressive primary surgery followed by platinum-based chemotherapy. Nevertheless, the risk of relapse is high, even in patients who achieve a complete response, and most of them develop platinum-resistant progressive disease, which restricts available therapeutic options. Despite impressive advances in surgical approaches and drugs, the 5-year survival for patients with advanced disease remains only about 30% [4]. Furthermore, although many efforts have been made to develop new prognostic markers, few molecular prognostic signatures have been developed, fewer have been validated, and clinically available today [5–9]. The highly lethal malignancy for EOC is mainly due to its propensity to form widespread peritoneal implants throughout the abdominal cavity [10].

Ovarian cancer cells detach from the primary tumor and float in the ascitic fluid as single cells or multicellular, chemo-resistant spheroids [11]. Then, floating cancer cells must resist anoikis triggered by lack of attachment to the mesothelium to evade clearance through the peritoneal lymphatics. Thus, metastatic progression is fueled by the mesothelium-tumor cells interactions, which, if successfully blocked, may promote the death and clearance of cancer cells [12]. Metastasizing EOC cells exhibit a two-step mode: first adhere to exposed mesothelial extracellular matrix (ECM) proteins, and then penetrate ECM under mesothelial cells [13]. The Urokinase Plasminogen Activator (uPA) and its specific Receptor (uPAR) are very important at multiple stages during development of intraperitoneal metastases of ovarian cancer cells [14–17]. uPAR is a glycosyl-phosphatidyl-inositol (GPI)-anchored receptor arranged in three domains (DI, DII, and DIII) linked by short sequence regions [18]. When expressed on cell surface, uPAR promotes cell-associated proteolysis by binding to uPA, which locally converts plasminogen into active plasmin, thus favoring tissue invasion and metastasis [14, 19]. Plasmin generated by uPA or uPA itself can cleave intact uPAR (DI-DIII), releasing DI, while the remaining GPI-anchored DII-DIII can remain on cell surface or be secreted in the extracellular milieu following cleavage of the anchor [20]. Indeed, full-length uPAR or fragments deriving from its cleavage on the cell surface may be released in soluble form in plasma and/or urine [21]. In EOC, overexpression of uPAR and the release of its truncated forms in sera and in the ascitic fluid, are associated with poor prognosis and unfavorable clinical outcome [17]. The domain boundary between DI-DII (uPAR84–95 sequence) is mainly projected on the external surface of uPAR, includes a protease-sensitive signaling region, and possesses a structural flexibility in both membrane-associated and soluble forms of uPAR [22, 23]. We and others have documented that uPAR84–95 sequence as well as the synthetic shorter pentapeptide uPAR88–92 (Ser-Arg-Ser-Arg-Tyr, SRSRY) act as potent regulators of cell migration and ECM attachment, independently of the uPA catalytic activity [14, 24–26]. Previous work from this laboratory showed that uPAR promotes ovarian cancer cell dissemination through its 84–95 sequence. Using hamster ovarian CHO-K1 cells lacking uPAR and stably transfected with cDNAs coding for uPAR containing or lacking the 84–95 sequence, we found that uPAR_84–95_ is indispensable for growth and intra-abdominal dissemination of ovarian cells orthotopically implanted in nude mice [27].

Mechanistically, uPAR84–95 and SRSRY exert these activities through their interaction with the Formyl Peptide Receptor type 1 (FPR1) which internalizes and activates the vitronectin receptor with an inside-out type of mechanism, involving PKC, AKT and MAPK signaling cascades [26, 28].

Human FPR1, originally identified in neutrophils, monocytes and macrophages, has been shown in recent years, to be expressed also in many non-myelocytic cells. Aberrant expression of FPR1 was detected in tumors of different origin and reported as a negative prognostic factor [29–31]. While the role of uPAR in favoring intra-abdominal dissemination of ovarian cancer cells is largely documented [17, 27], no data regarding expression and possible role of FPR1 on the surface of ovarian cancer cells are currently available. In the past years, we generated synthetic peptides containing the substitution of Ser90 with a glutamic acid or a α-*amino-isobutyric acid* residue in the Ser^88^-Arg-Ser-Arg-Tyr^92^ sequence inhibiting the uPAR/FPR1 interaction, directional cell migration, invasion and angiogenesis [32–35]. Later, to improve their chemical stability and half-life, we developed a new library of retro-inverso peptides [36]. The lead compound Ac-(D)-Tyr-(D)-Arg-Aib-(D)-Arg-NH_2_ (RI-3) is stable in human serum, adopts the turn structure typical of uPAR/FPR1 antagonists, and competes with fMLF and SRSRY for binding to FPR1, preventing SRSRY-induced FPR1 internalization as well as p38 MAPK and PI3K/AKT signaling cascades [36], which are documented to mediate FPR1 signal transduction pathways [30]. Interestingly, RI-3 inhibits migration and invasion of sarcoma and melanoma cells in a dose dependent manner, an overall 50% reduction of cell migration and invasion being reached in the picomolar and nanomolar range, respectively [36, 37]. Recently, to understand the structural basis of the RI-3 inhibitory effects, the FPR1/fMLF, FPR1/SRSRY and FPR1/RI-3 complexes were modeled and analyzed, focusing on the binding pocket of FPR1 and the interaction between the amino acids that signal to the FPR1 C-terminal loop. We found that RI-3 shares the same binding site of fMLF and SRSRY on FPR1. However, while fMLF and SRSRY display the same agonist activation signature, RI-3 does not interact with the activation region of FPR1, keeping receptor anchored on cell membrane and hence unable to internalize and activate signaling, [38]. In this study, we analyzed the expression of FPR1 in tissues from patients affected by EOC. Then, by using primary EOC cells, we analyzed the role of uPAR/FPR1 crosstalk enabling cancer cells to adhere onto matrices and mesothelial cell monolayers. We also show that RI-3 successfully prevents the capability of ovarian cancer cells to adhere onto vitronectin and invade mesothelium.

## Methods

### EOC cell line, EOC primary cultures and transfection

Human ovarian carcinoma SKOV-3 and A2780 cell lines, obtained from the Cell Factory of the National Cancer Institute of Genova, were cultured in DMEM or RPMI, respectively, supplemented with 10% heat-inactivated fetal bovine serum (FBS), penicillin (100 μg/mL), streptomycin (100 U/mL) and maintained at 37 °C in a humidified atmosphere of 5% CO_2_. To obtain primary cultures, a representative sample from the EOC excision (*∼*1 cm *×* 1 cm) of consenting patients (Table 1) was immediately minced by scalpel under sterile conditions, and incubated with 1.0 mg/mL collagenase XI (Sigma) for 3 h at 37 *°*C under gentle agitation, as previously described [39]. Cells, recovered by centrifugation at 1500 rpm, were cultured in 6-well multi-dish plates in DMEM with the addition of 10% FBS, 100 IU/mL penicillin and 50 μg/mL streptomycin. Isolated cell clusters were further amplified in growth medium until an adherent, homogeneous cell population was obtained. The epithelial phenotype was identified by the positive staining for the CD326 pan-epithelial differentiation antigen (Miltenyi Biotec)**.**

**Table 1:** Clinicopathologic findings, uPAR and FPR1 expression in EOC tissues

Patients	Age	Site	Size (cm)	Histology	FIGO	uPAR score	FPR1 score
1	64	right ovary	10x10x8	HGSC	III	2	1
2	44	left ovary	11x9x6.5	HGSC	IV	1	2
3	62	left ovary	2.5x1x1	HGSC	III	1	2
4	62	left ovary	4x3x2	HGSC	III	2	2
5	49	left ovary	4x2x0.5	HGSC	III	2	2

SKOV-3 and primary EOC cells were stably transfected with cDNA coding for the Green Fluorescent Protein (GFP) using pEGFP-N1 vector (Clontech) and polyfectamine transfection reagent (Quiagen) as described [36]. Transfected cells were selected by Geneticin at 0.8 mg/mL for 15 days, pooled and cultured in the presence of 0.5 mg/mL Geneticin. G418-resistant cells expressing the highest levels of GFP were isolated and amplified.

### Flow cytometry

Cells (0.5 × 10^6^ cells/sample) were detached using 200 mg/L EDTA, 500 mg/L trypsin (Cambrex) and incubated with 100 μl phosphate buffered saline (PBS) containing diluents (CTRL), 10 μl uPAR (CD87)-APC-conjugated antibody **(**Miltenyi Biotec), or 30 μl FPR1 PE-conjugated Antibody **(**R&D Sistems) for 10 min at 4 °C and 30 min at 23 °C, respectively. After extensive washing with PBS, cells were re-suspended in 0.4 ml PBS and analyzed by flow cytometry using the BD FACSCanto II (BD Biosciences) and the FlowJo software. Control staining was performed using FITC-conjugated isotype-mached mouse IgG.

### Primary culture of mesothelial cells

Human peritoneal mesothelial cells (HPMCs) were isolated and characterized as previously described [27]. Briefly, specimens of human omentum were obtained from consenting patients undergoing elective abdominal surgery (~ 2 cm^2^). Blunt dissection removed excess fat and provided predominantly transparent samples of the tissue. The omentum was washed several times with sterile PBS to remove any contaminating red blood cells and specimens were subjected to disaggregation with 5 mL of 0.125% (wt/vol) trypsin, 0.01% (wt/vol) EDTA (Sigma) for 20 min at 37 °C with continuous rotation. Then, cell suspension was centrifuged at 800 × g for 5 min, the cell pellet was washed once in F12 culture medium containing 10% FBS, suspended in the same medium to a volume of 5 mL and seeded in 25 cm^2^ tissue culture flasks. Half the medium was changed 24 h later and fully replaced once every 3 days. The mesothelial phenotype was identified by the uniform cobblestone appearance at confluence, by the lack of staining for factor VIII-related antigen, and by the positive staining for cytokeratin 8 and 18 (Clone EP17/EP30) and vimentin, all purchased by Dako.

### Peptides

The peptides SRSRY, ARARY and Ac-(D)-Tyr-(D)-Arg-Aib-(D)-Arg-NH2 (RI-3) were custom-synthesized by JPT Peptide Technologies, Germany. Peptides were synthesized on solid-phase with Fmoc/t-Bu chemistry and purified by reversed-phase HPLC using water/acetonitrile gradients, and characterized by UPLC-MS. N-Formyl-L-methionyl-L-leucyl-L-phenylalanine (fMLF) and the fluorescein conjugate hexapeptide Formyl-Nle-Leu-Phe-Nle-Tyr-Lys (FITC-fMLF) peptides were purchased by Invitrogen and Molecular Probes, respectively. The fluorescein-conjugate Ac-(D)-Tyr-(D)-Arg-Aib-(D)-Arg-Ahx-Lys (N^ɛ^-FITC)-NH_2_ (FITC-RI-3) peptide was custom-synthesized by MicroGem, Italy.

### Western blot

Cells detached using 200 mg/L EDTA, 500 mg/L trypsin (Cambrex), were lysed in RIPA buffer (10 mM Tris pH 7.5, 140 mM NaCl, 0.1%SDS, 1% Triton X-100, 0.5% NP40) containing protease inhibitor mixture. Protein content of cell lysates was measured by a colorimetric assay (BioRad). 30 and 60 μg proteins were separated on 10% SDS-PAGE under unreducing (to detect uPAR) or reducing conditions (to detect FPR1) and transferred onto a polyvinylidene fluoride membrane. Membranes were blocked with 5% non-fat dry milk and probed with 1 μg/mL R4 anti-uPAR mAb, recognizing the DII-DIII uPAR domains, 1 μg/mL anti-FPR1 polyclonal antibody (Ab) (#128296 Ab, Abcam), or 0.2 μg/mL GAPDH Ab (Santa Cruz Biotechnology). Washed filters were incubated with horseradish peroxidase-conjugated anti-mouse or anti-rabbit antibody and detected by ECL (Amersham- GE Healthcare). Densitometry was performed using the NIH Image 1.62 software (Bethesda, MD.

### Adhesion of EOC cells onto extracellular matrix proteins

Adhesion assays were performed using 24 multi-well plates coated with 1% BSA, 5 μg mL vitronectin (Vn), 5 μg mL fibronectin (Fn), 5 μg mL laminin (Lm) or 15 μg mL collagen (Coll) diluted in serum-free medium. Sub-confluent cells were harvested by a mild trypsinization, incubated with 10% FBS / DMEM for 1 h at 37 °C, 5% CO_2_, acid treated with a buffer containing 50 mM glycin-HC1, 0.1 M NaCl, pH 3 for 4 min at 22 °C, to remove receptor-bound ligands [40], and counted. Viable cells (2 × 10^5^ cells per well) were plated in serum-free medium, in the absence or presence of 2 μg/mL R3 mAb recognizing the DI uPAR domain, 2 μg/mL399 anti-uPAR (American Diagnostica), 2 μg/mL anti-uPAR84-95 (PRIMM), 2 μg/mL anti-α-tubulin (Cell Signaling) Abs or 10 nM RI-3, and allowed to adhere for 2 h at 37 °C, 5% CO_2_. A subset of experiments was performed using cells desensitized with 100 nM SRSRY, 100 nM fMLF to block FPR1 activity as described [28], or 100 nM control peptide ARARY for 30 min at 37 °C in humidified air with 5%CO_2._ At the end of the assay, adherent cells were removed with 0.05% trypsin, and counted. The number of adherent cells was expressed as a percentage of the basal cell adhesion (CTRL). The experiments were performed at least three times in triplicate.

### Adhesion kinetic of EOC cells monitored in real time

This assay was performed using E-16-well plates and the xCELLigence Real Time Cell Analysis technology. Bottom wells were coated with serum-free medium, 5 μg mL Vn, 5 μg mL Fn, 5 μg mL Lm or 15 μg mL Coll diluted in serum-free medium prior to seeding cells (1 × 10^4^ cells/well) suspended in in serum-free medium. Cells that adhere to the bottom of plates cause impedance changes which are proportional to the number of cells. The impedance value of each well was automatically monitored in real-time for at least 2 h and expressed as a cell index value. The experiments were performed three times in quadruplicate.

### EOC cell adhesion onto mesothelium

HPMCs (1 × 10^5^ cells/well) suspended in growth medium, were seeded in 24-well plates and allowed to adhere for about 20 h until they form a confluent monolayer, prior to seeding GFP-tagged EOC cells suspended in growth medium plus diluents or 10 nM RI-3. At the indicated times, plates were accurately washed with PBS and cell-associated fluorescence was assessed by a fluorescence plate reader a fluorescence plate reader (Victor 3, Perkin Elmer), using 485 nm excitation and 535 nm emission filters. The experiments were performed three times in triplicate.

### HPMC invasion by EOC cells monitored in real time

HPMCs (5 × 10^3^ cells/well) suspended in growth medium, were seeded in E-16-well plates and allow to adhere for 18–20 h until they form a confluent monolayer [27], prior to seeding EOC cells (2 × 10^4^ cells/well) in growth medium plus/minus 10 nM RI-3. When HPMCs are challenged with invading cells, there is a drop in electrical resistance within 2–4 h which is monitored in real-time as the Cell Index changes due to the rupture of mesothelium. The experiments were performed three times in quadruplicate.

### Fluorescence microscopy

Cells (2 × 10^4^/sample) were seeded on glass coverslips and cultured for 24 h in growth medium. Then, slides were washed with PBS, fixed with 2.5% formaldehyde in PBS for 10 min at 4 °C and incubated for 1 h at 4 °C with 2 μg/mL R4 anti-uPAR mAb or 1:100 rabbit anti-FPR1 Ab (#113531Ab, Abcam). Then, 1:700 goat Alexa Fluor 488 anti-rabbit IgG, or rabbit Alexa Fluor 488-conjugated F(ab’)2 fragment of anti-mouse IgG (Molecular Probes) were applied to slides at 23 °C for 40 min. Nuclear staining was performed with 4-6-diamidino-2-phenylindole dye (DAPI). To analyze agonist-dependent FPR1 internalization, cells grown on glass slides were exposed to 10 nM FITC-fMLF or 10 nM FITC-RI-3 diluted in serum-free DMEM for 30 min at 37 °C as described [32, 38]. After extensive washing with PBS, coverslips were mounted using 20% (w/v) mowiol, visualized with the 510 META-LSM confocal microscope (Carl Zeiss).

### Ligand binding assay

EOC cells were pre-incubated with diluents or the indicated unlabeled peptides for 60 min at 4 °C (to avoid internalization), extensively rinsed with phosphate buffer saline (PBS), exposed to 10 nM FITC-RI-3 diluted in PBS, for additional 60 min at 4 °C and again rinsed with PBS. Quantification of cell associated fluorescence was assessed by reading cells with the fluorescence plate reader Victor 3. The experiments were performed three times in duplicate.

### EOC patients

Forty-two patients admitted to the National Cancer Institute of Naples were recruited in the study. All patients provided written informed consent for the use of samples according to the institutional regulations and the study was approved by the ethics committee of the National Cancer Institute of Naples. All EOC cases were reviewed according to 2014 WHO classification criteria [41], using standard tissue sections and include: *n* = 3 low-grade serous carcinoma (LGSC), *n*=33 high-grade serous carcinoma (HGSC), *n*=5 ovarian clear cell carcinoma (OCCC), and *n*=1 mucinous ovarian carcinoma (mEOC) tissues. Medical records have been reviewed for clinical information, including histologic parameters assessed on standard H&E-stained slides.

### TMA building

A tissue microarray (TMA) was built using 37 EOC tissue samples, including 17 primary and matched metastatic tissues. Tissue cylinders with a diameter of 1 mm were punched from morphologically representative tissue areas of each ‘donor’ tissue block and brought into one recipient paraffin block (3 × 2.5. cm) using a semi-automated tissue array (Galileo TMA).

### Immunohistochemistry

Immunohistochemistry (IHC) was carried out on slides from formalin-fixed, paraffin-embedded tissues, in order to evaluate the expression of uPAR (on 5 HGSC cases) and FPR1. Paraffin slides were deparaffinized in xylene and rehydrated through graded alcohols. Antigen retrieval was performed with slides heated in 0.01 M citrate buffer (pH 6.0.) in a bath for 20 min at 97 °C. After antigen retrieval, the slides were allowed to cool, rinsed with Tris buffered saline (TBS) and the endogenous peroxidase was inactivated with 3% hydrogen peroxide as described [42, 43]. After protein block (5% BSA in PBS), the slides were incubated overnight at 4 °C with 1 μg/mL rabbit anti-human FPR1 polyclonal Ab (cod. NLS2086, Novus Biologicals) or the R4 anti-uPAR mAb, recognizing the DIIDIII uPAR domains (Dako). Then, sections were incubated with mouse biotin anti-rabbit or goat anti-mouse secondary IgG Abs for 30 min. Immunoreactivity was visualized by means of avidin-biotin-peroxidase complex kit reagents (Novocastra, Newcastle, UK) and the chromogenic substrate. Finally, sections were counterstained with hematoxylin and mounted. Appropriate inner cells were considered as controls. For each antibody, cytoplasmic and membrane staining were recorded. The uPAR and FPR1 staining intensity of epithelial tumor cells were graded as faint (score 0), moderate (score 1), or intense (score 2). TMA tissues were scored semi-quantitatively by evaluating the proportion of positive tumor cells over the total number of tumor cells (percentage of positive tumor cells/tissue microarray punch). Negative/low and positive high expressing tissues were recorded when neoplastic cells expressing FPR1 were between 0 and 10%, and ≥ 80%, respectively. All sections were evaluated in a blinded fashion by 2 investigators.

### Statistical analysis

The results are expressed as the means ± standard deviations of the number of the indicated determinations. Data derived from in vitro experiments were analyzed by one-way ANOVA and post hoc Dunnett t-test for multiple comparisons. *P* < 0.05 was accepted as significant. Pearson’s Chi-square (χ^2^) test was used to analyze the correlations between FPR1 expression and clinicopathologic parameters. Data analysis and summarization were conducted using the SPSS 20.0 software (SPSS Inc., Chicago, Illinois USA).

## Results

### Requirement of uPAR84–95 and FPR1 for the adhesion of SKOV-3 cells onto vitronectin

**Fig. 1:**
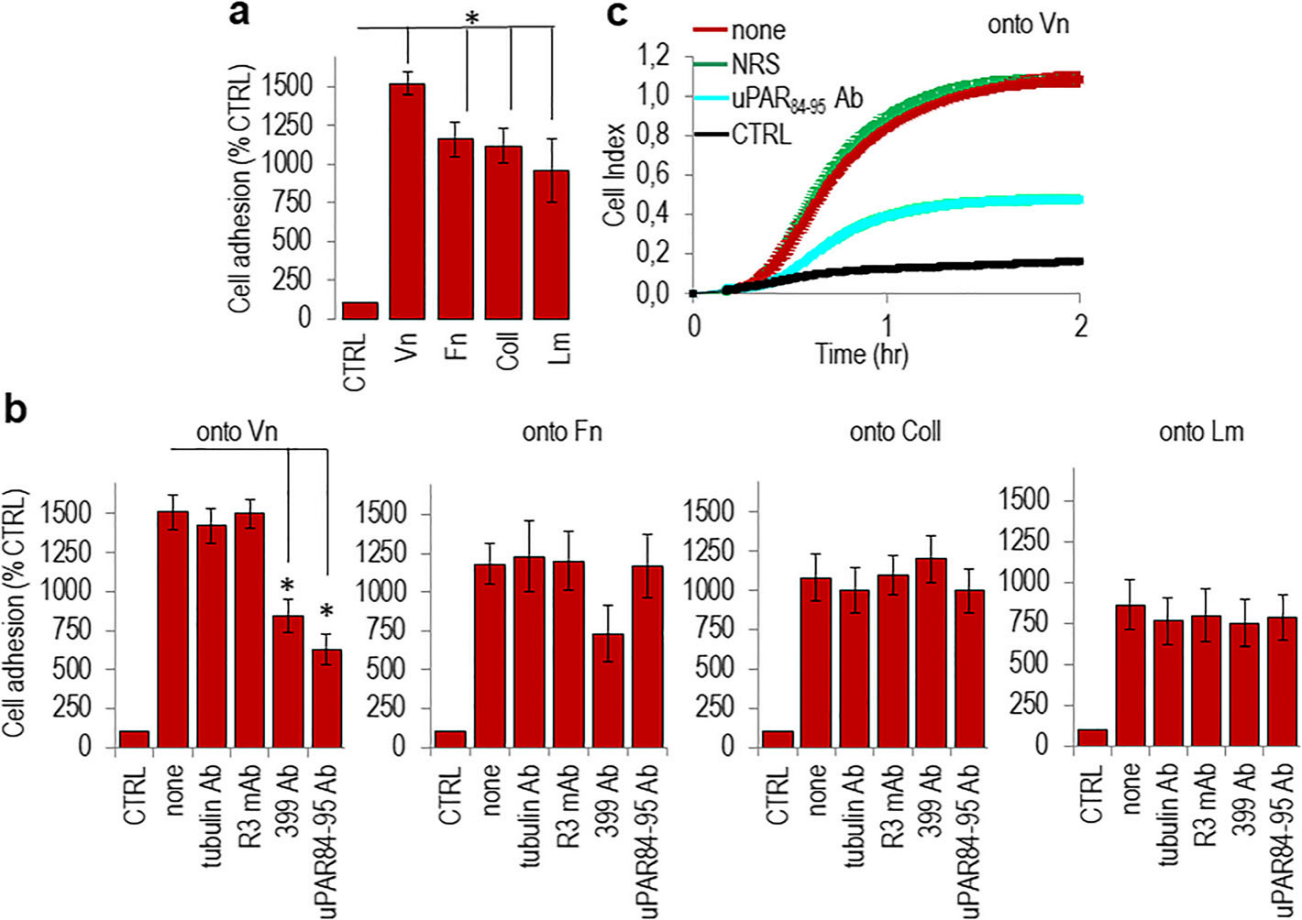
Requirement of the uPAR84–95 sequence for SKOV-3 cell adhesion onto vitronectin. **a.** Viable SKOV-3 cells (2 × 10^5^ cells/well) were seeded in triplicate in 24-well plates coated with 1% BSA (CTRL), 5 μg mL vitronectin (Vn), 5 μg mL fibronectin (Fn), 5 μg mL laminin (Lm) or 15 μg mL collagen (Coll) and allowed to adhere for 2 h. The number of adherent cells was expressed as a percentage of CTRL. Data are the means ± SD of three independent experiments with *P < 0.001. **b.** Cell adhesion of SKOV-3 cells in 24-well plates coated with 1% BSA (CTRL) or indicated matrix proteins in the absence (none) or presence of 2 μg/ml R3 anti-uPAR mAb, 2 μg/ml 399 anti-uPAR Ab, 2 μg/ml anti-uPAR84–95 Ab, or 2 μg/ml anti-α-tubulin Ab for 2 h. The number of adherent cells was expressed as a percentage of CTRL. Data are the means ± SD of three independent experiments with *P < 0.001. **c.** Adhesion of SKOV-3 cells onto Vn monitored by the xCELLigence system. Cells (1 × 10^4^ cells/well) suspended in serum-free medium (CTRL) with or without 2 μg/ml anti-uPAR84–95 or 2 μg/ml normal rabbit serum (NRS) were seeded on Vn-coated E-plates. The impedance value of each well was automatically monitored in real-time for 2 h and expressed as a cell index value. Data represent mean ± SD from a quadruplicate experiment representative of 3 replicates

**Fig. 2:**
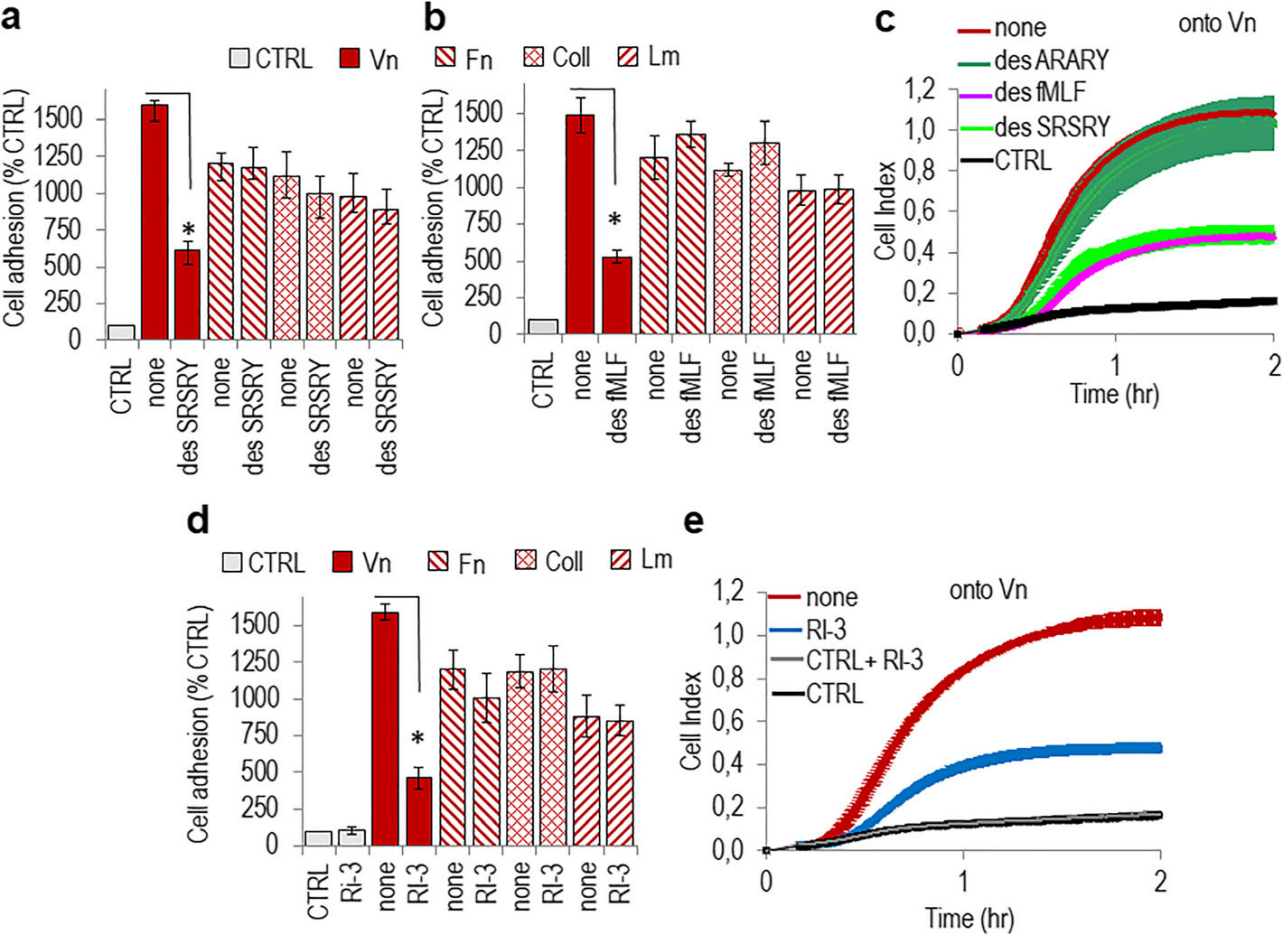
FPR1 requirement for adhesion of SKOV-3 cells onto vitronectin. **a-b.** Cell adhesion of SKOV-3 cells pre-incubated with serum-free medium (none), 100 nM SRSRY (**a**) or 100 nM fMLF (**b**) for 37 min at 37 °C in humidified air with 5%CO_2,_ onto the indicated matrix proteins. Cell adhesion was expressed as percentage of CTRL (cells adherent to plated coated with 1% BSA). Data are the means ± SD of three independent experiments with *P < 0.001. **c.** Adhesion onto Vn of SKOV-3 cells desensitized with diluents (none), 100 nM SRSRY, 100 nM fMLF, or 100 nM ARARY, monitored in real time for 2 h by the xCELLigence system. Data represent mean ± SD from a quadruplicate experiment representative of 3 replicates. **d.** Adhesion of SKOV-3 cells onto the indicated matrix proteins in the absence or presence of 10 nM RI-3. The number of adherent cells was expressed as a percentage of CTRL (cells adherent to plated coated with 1% BSA). Data are the means ± SD of three independent experiments with *P < 0.001. **e.** Adhesion of SKOV-3 cells onto Vn in the absence or presence of 10 nM RI-3, monitored in real time for 2 h by the xCELLigence system. Data represent mean ± SD from a quadruplicate experiment representative of 3 replicates

Metastasizing EOC cells attach to the peritoneum through the interaction with exposed mesothelial ECM proteins [13]. We investigated the specific contribution of the uPAR84–95 chemotactic sequence in regulating the adhesion of SKOV-3 human ovarian carcinoma cells to the ECM proteins. Adhesion assays were performed by plating cells onto vitronectin (Vn)-, collagen (Coll)-, fibronectin (FN)-, laminin (LM)- or BSA- coated wells, the last as control (CTRL). SKOV-3 cells, expressing a considerable amount of uPAR and FPR1 [27], were able to adhere to all tested ECM proteins**,** although at a different extent (Fig. 1a). However, as already documented [35], only cell adhesion onto Vn is uPAR 84–95-dependent because it is reduced to the basal level by cell pre-exposure to #399 anti-uPAR as well as by anti-uPAR84–95 Abs. In contrast, the R3 anti-uPAR mAb, recognizing the D1 uPAR domain or control anti-tubulin Ab were ineffective (Fig. 1b). Similar results were obtained when SKOV-3 cell adhesion onto Vn was monitored in real time for 2 h, showing that adherence to Vn is prevented by anti-uPAR84–95 Ab, but not by normal rabbit serum (Fig. 1c). The involvement of FPR1 was tested by desensitizing cells with 100 nM fMLF (Fig. 2a) or 100 nM SRSRY (Fig. 2b) for 30 min at 37 °C, leading to unavailability of FPR1 on cell surface [37, 38]. Desensitized cells lose the ability to adhere onto Vn but not onto Coll, Fn or Lm (Fig. 2a–b). Real time assessment of cell adhesion onto Vn, allowed us to confirm that pre-exposure to an excess of fMLF or SRSRY abrogates cell capability to adhere onto Vn, whereas the ARARY control peptide is ineffective (Fig. 2c). To further analyse the role of uPAR/FPR1 complexes in favoring cell adhesion onto Vn, SKOV-3, cells were allowed to adhere onto Vn in the presence/absence of the retroinverso peptide RI-3 that binds FPR1 keeping it anchored to cell membrane and incapable to function [38]. We found that 10 nM RI-3 neither affected basal cell adhesion, nor cell adhesion to Coll- Fn- and Lm-coated plates, but dramatically reduced the extent of cell adhesion onto Vn (Fig. 2d–e). Confirmatory experiments were performed using A2780 human ovarian carcinoma cells which neither express uPAR nor FPR1 on cell surface, as shown by Fax analysis (Additional file 1: Figure S1a). A2780 cells adhere at a different extent onto Coll, Fn or Lm, but they lack adhesion to Vn, as expected on the basis of the missing uPAR84–95 and FPR1 receptors. As expected, in the absence of the target receptor, RI-3 peptide was ineffective (Additional file 1: Figure S1b).

### RI-3 prevents mesothelium invasion by SKOV-3 ovarian cancer cells

**Fig. 3:**
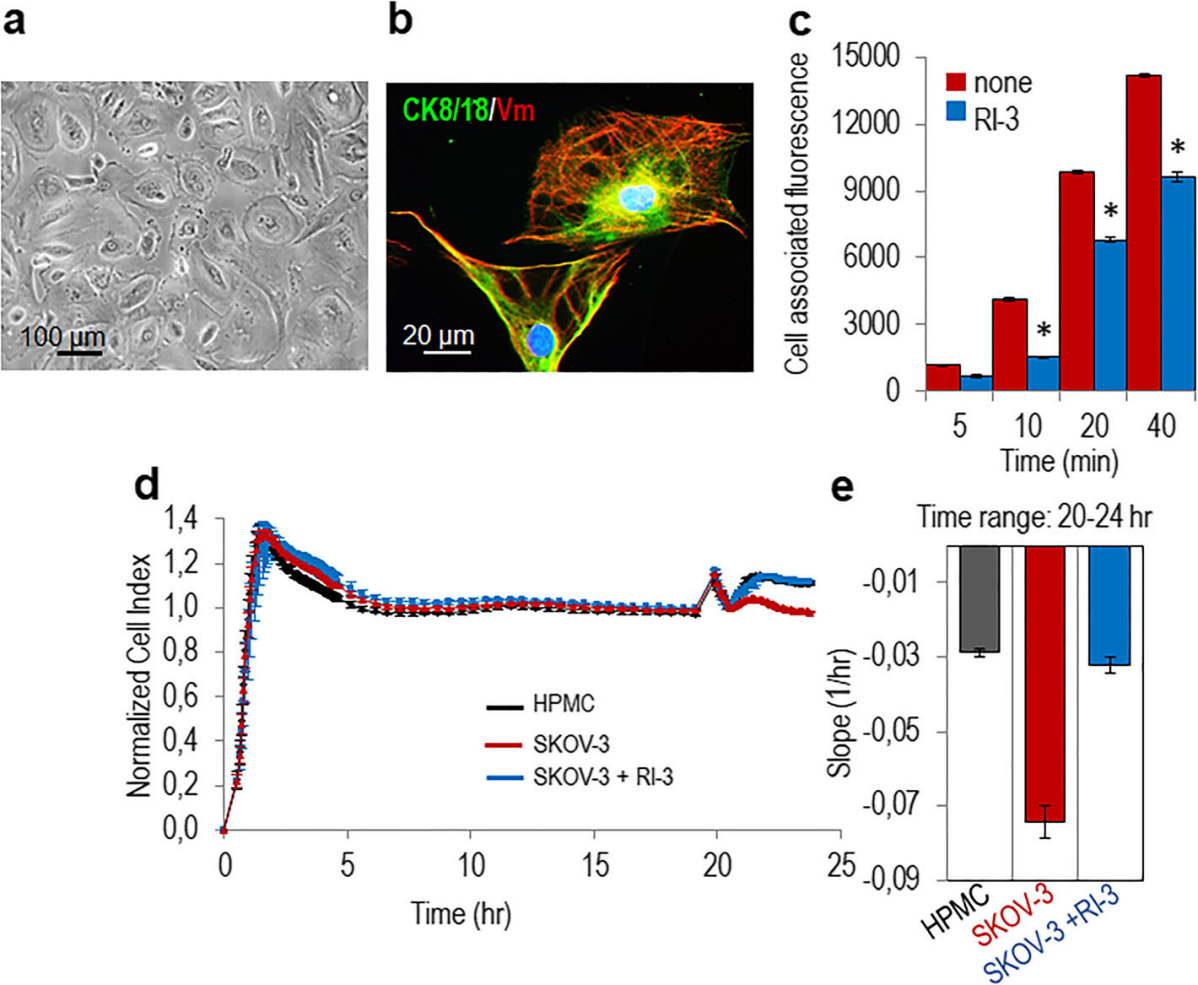
RI-3 prevents mesothelial cell adhesion and invasion by SKOV-3 cells. HPMCs were purified from human omental specimens and cultured as described in the Methods. **a-b**. Representative images of HPMCs visualized by phase contrast microscopy (**a**) or stained for cytokeratin 8/18 (green) and vimentin (red) (**b**). Nuclei were stained blue with DAPI. Original magnification: 400x (**a**), 1000 x (**b**). **c.** HPMCs were seeded in 24-well plates and allowed to attach and form a monolayer for about 20 h prior to seeding GFP-SKOV-3 cells suspended in complete medium plus diluents (none), or 10 nM RI-3 at 37 °C, 5% CO_2_. At the indicated times, cell associated fluorescence was assessed by a fluorescence plate reader. Data represent means ± SD of three independent experiments performed in triplicate with *P < 0.0001 vs none. **d-e.** Mesothelium invasion by SKOV-3 cells. HPMCs (5 × 10^3^ cells/well) were seeded in E-16-well plates in growth medium and allow to adhere for 20 h until they form a confluent monolayer (plateau curves). Then, SKOV-3 cells (2 × 10^4^ cells/well) suspended in growth medium plus/minus 10 nM RI-3 were seeded on mesothelial cell monolayer. The invasion of mesothelium by SKOV-3 cells was monitored in real-time as changes in Cell Index due to breaking of the monolayer integrity (**d**). Slopes represent the change rate of Cell Indexes generated in a 20–24 h time frame (**e**). Data represent mean ± SD from a quadruplicate experiment representative of 3 replicates

Using hamster ovarian CHO-K1 cells engineered to expose or lacking the 84–95 sequence, we have previously documented that the uPAR_84–95_ is required for cell capability to adhere to and cross mesothelial cell monolayers [27]. To ascertain if RI-3 peptide may affect SKOV-3 ovarian cell adhesion, mesothelial cells were purified from human omental specimens as previously described [27]. Pure mesothelial cells (HPMCs) were identified by their cobblestone appearance at semi-confluence (Fig. 3a), and because 87% of cells appeared positive for cytokeratin 8/18 (green) and vimentin (red) (Fig. 3b). Mesothelial cells were allowed to form a monolayer and Green Fluorescent Protein (GFP)-tagged SKOV-3 cells were seeded on top in the presence/absence of 10 nM RI-3. At the indicated times, non-adherent cells were removed and the cell-associated fluorescence was measured using a fluorescence plate reader. GFP-SKOV-3 cells adhere early to mesothelium: already after 5–10 min, we found an appreciable cell adhesion to mesothelium, that increased with time. After 5, 10, 20, and 40 min, 10 nM RI-3 reduced cell associated fluorescence by 57, 47, 58, and 54%, respectively (Fig. 3c), indicating that the attachment of SKOV-3 cells to mesothelium is prevented by the RI-3 peptide. To investigate whether, RI-3 peptide also reduces mesothelium invasion by ovarian cancer cells, the ability of SKOV-3 cells to cross mesothelial cell monolayers was analyzed in the presence or the absence of RI-3, using the xCELLigence technology. As expected, an appreciable reduction of mesothelium integrity was achieved by SKOV-3 cells. In contrast, 10 nM RI-3 abrogated the capability of SKOV-3 cells to disrupt mesothelial cell monolayer (Fig. 3d–e). In contrast, A2780 cells exhibited a poor ability to cross mesothelium and RI-3 added at 10 nM concentration was ineffective (Additional file 1: Figure S1c). Taken together, these data indicate that the RI-3 peptide reduces SKOV-3 cell adhesion to vitronectin and mesothelial cell monolayers, both processes being sustained by the uPAR84–95/FPR1 crosstalk.

### Co-ordinated uPAR and FPR1 expression in human ovarian cancer tissues

**Fig. 4:**
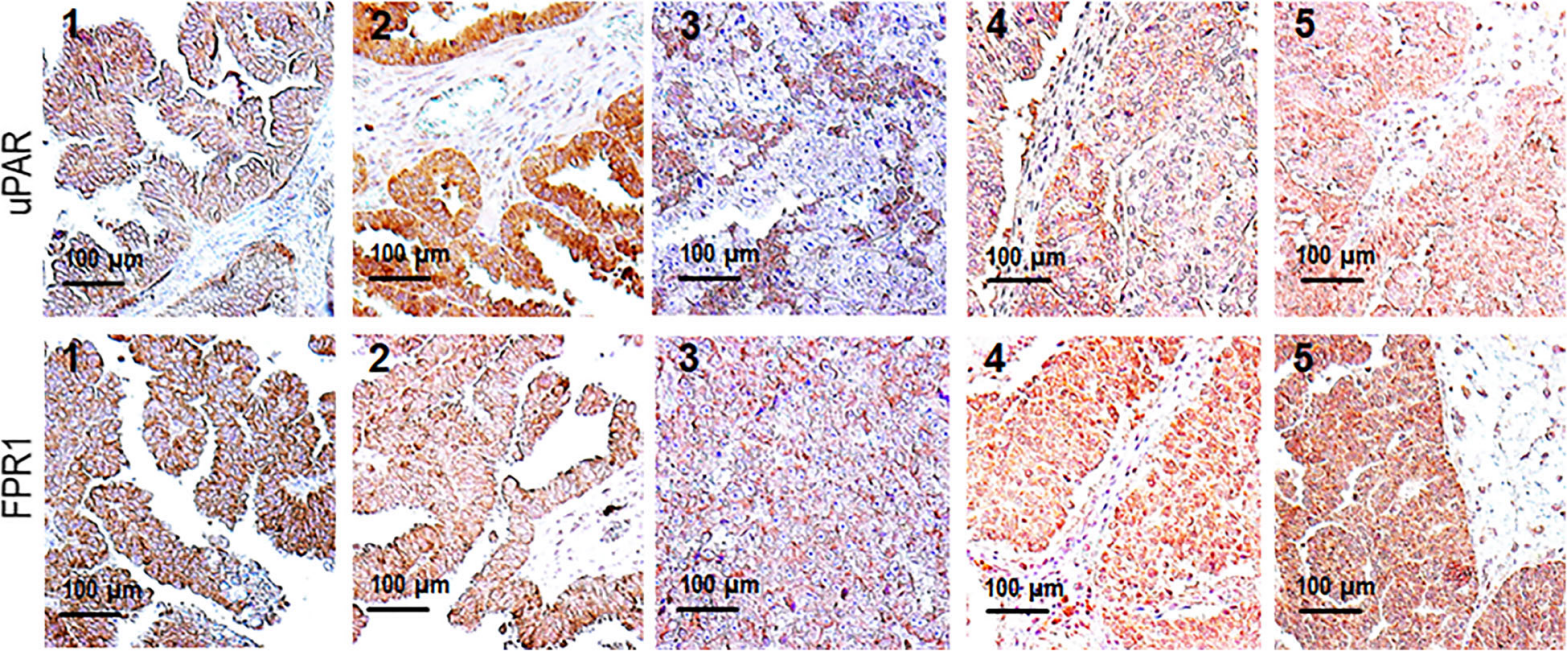
uPAR and FPR1 co-ordinated expression in human EOC tissues. Expression of uPAR and FPR1 in 5 HGSC tissues. Representative images of formalin-fixed, paraffin embedded sections subjected to immunohistochemical staining with R4 anti-uPAR mAb or anti-FPR1 Ab. Nuclei were stained blue with hematoxylin. Original magnification: × 200

While the uPAR role in promoting intra-abdominal dissemination of ovarian cancer cells is largely documented [17], to the best of our knowledge, the expression of FPR1 in EOC tissues has never been reported. Thus, we analyzed the uPAR and FPR1 expression on formalin-fixed paraffin-embedded sections from five serous adenocarcinoma tissues by IHC. Table 1 reports clinical data, pathological findings and FIGO staging of *n* = 5 HGSC cases. The uPAR and FPR1 staining intensity of epithelial tumor cells was graded as faint (score 0), moderate (score 1), or intense (score 2) (Table 1) and representative patterns are shown in Fig. 4. Except for the HGSC case #3 showing only cell clusters reactive to R4 anti-uPAR mAb, all tumors exhibit a diffuse, heterogeneous pattern of staining, mainly localized on tumor plasma cell membranes (Fig. 4). A diffuse staining of the epithelial tumor cells using anti-FPR1 Ab was obtained in all sections, with a prominent staining of plasma cell membranes, often observed in positive tumor cells. No apparent detection of either uPAR and FPR1 was appreciated in intra-or peri-tumoral stroma with the exception of FPR1 expressing macrophages as already documented [44]. According to Ahmed and colleagues, we found no expression of uPAR in normal ovarian tissues [45], while FPR1staining of ciliated epithelium of healthy fallopian tubes adjacent to neoplastic areas was observed only in the HGSC case #4 *(not shown*).

**Fig. 5:**
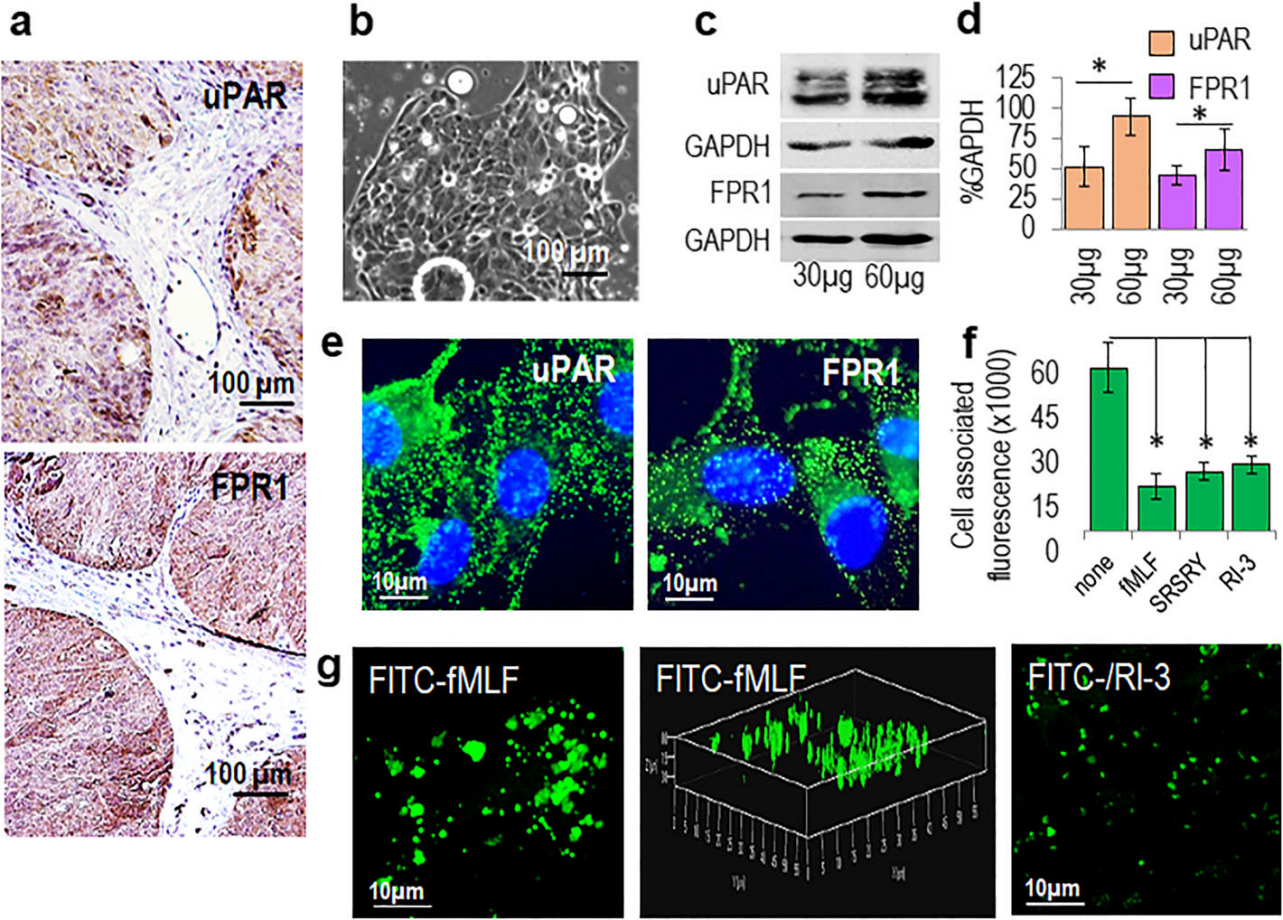
Generation of a primary cell culture from a human high-grade *serous ovarian cancer*. **a** HGSG *tissue* (patient # 5) processed for IHC analysis of uPAR and FPR1 expression on paraffin sections. Nuclei were stained blue with hematoxylin. Original magnifications: × 100. **b** Primary EOC cells derived from the same HGSG *tissue* visualized by phase contrast microscopy. Original magnification: × 400. **c** Western blotting of whole cell lysate from EOC cells with R4 anti-uPAR mAb, anti-FPR1 Ab, and anti-GAPDH Ab. **d** Bar graphs showing the average quantification of the uPAR/GAPDH and FPR1/GAPDH content from 3 independent experiments. Statistical significance with *P < 0.001. **e** Images of human EOC cells immunostained with R4 anti-uPAR mAb or anti-FPR1 Ab and visualized by a fluorescence inverted microscope. Nuclei were stained blue with DAPI. Original magnification: 1000x. **f** Fluorescence associated to EOC cells pre-incubated with diluents (none), 100 nM fMLF, 100 nM SRSRY or 100 nM RI-3, for 60 min at 4 °C, and then exposed to 10 nM FITC-RI3 for additional 60 min at 4 °C. Data represent mean +/− SD from three experiments performed in triplicate with * *P* < 0.001. **g** EOC cells exposed to 10 nM FITC-fMLF or 10 nM FITC-RI-3 for 30 min at 37 °C and then visualized using a Zeiss 510 Meta LSM microscope in 2D (left) or 3D (right) projections. Original magnification: 630×

In order to obtain primary cultures of EOC cells, representative fragments of tissue samples were fragmented and cells recovered, as described in the Methods. By enzymatic digestion of a HGSC tissue sample (patient #5), a primary cell culture expressing high levels of both uPAR and FPR1 on cell surface as shown by IHC, was obtained (Figs. 4 and 5a). Subsequent sub-cloning of isolated cell clusters (Fig. 5b) followed by five passages allowed us to obtain an adherent, homogeneous, CD326 positive cell population. These EOC cells express a considerable amount of both uPAR and FPR1 as shown by Western blot analysis of cell lysates and immunofluorescence experiments with Anti-uPAR mAb and anti-FPR1 Ab (Fig. 5c, d, e) and Additional file 1: Figure S2 for full blots). Competition binding assays with FITC-RI-3 that we have previously reported binding FPR1 and inhibiting cell migration like the non-fluorescent peptide [38], revealed a specific, saturable RI-3 uptake on EOC cell surface, which was prevented by either SRSRY and fMLF peptides (Fig. 5f). As expected, when binding experiments were carried out at 37 °C, FITC-fMLF caused FPR1 internalization as revealed by punctuate green fluorescent intra-cytoplasmic spots which were only slightly detectable in cells exposed to FITC-RI-3, indicating little or no internalization (Fig. 5g). These findings indicate that a uPAR/FPR1 crosstalk does occur on the surface of primary ovarian cancer cells and that, as already documented in FPR1 overexpressing rat basophilic leukaemia RBL-2H3/ETFR cells [38], RI-3 binds to FPR1 blocking its internalization and function.

### RI-3 inhibits primary EOC cell adhesion onto vitronectin and mesothelium invasion

**Fig. 6:**
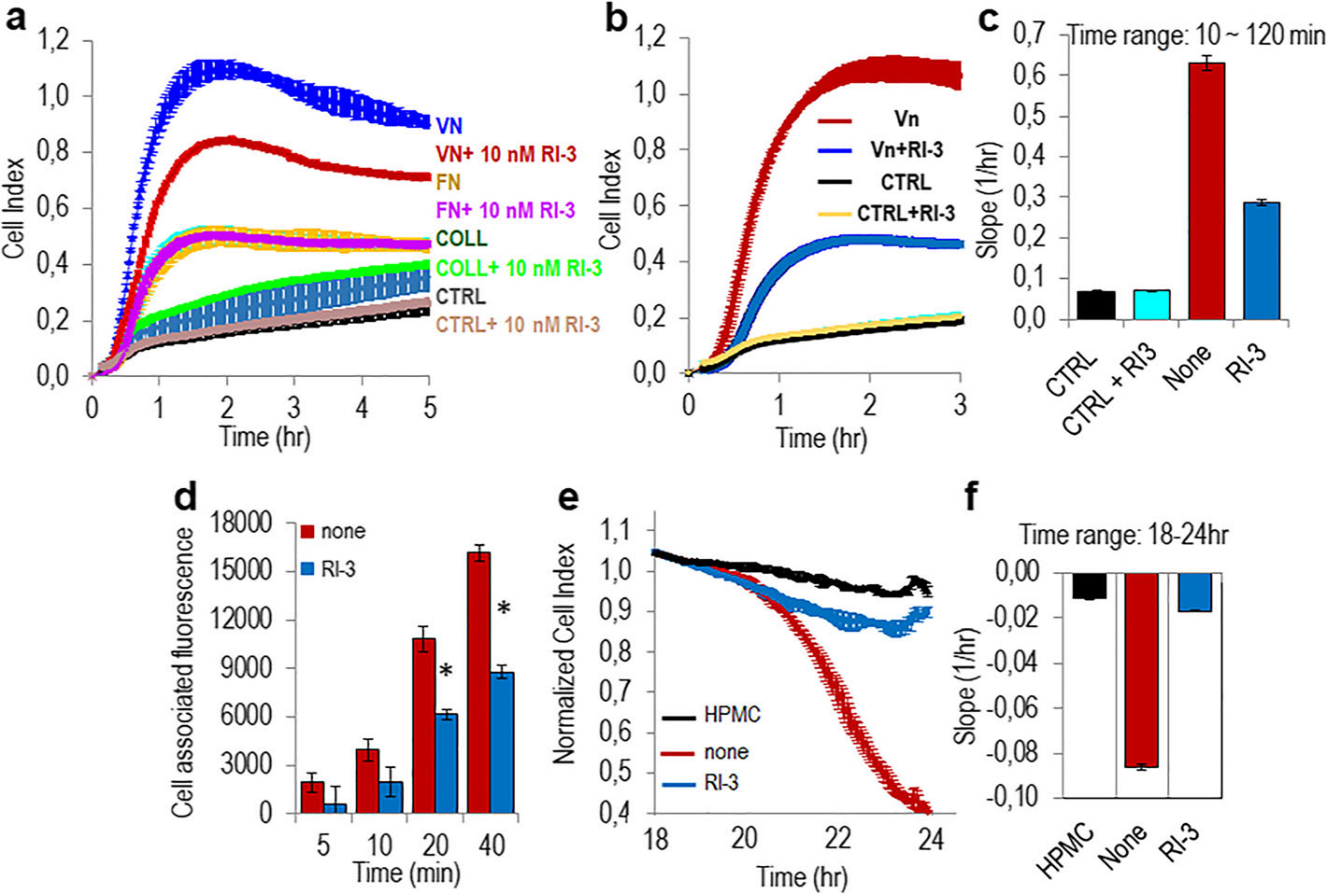
RI-3 prevents EOC cell adhesion and subsequent invasion of mesothelium. **a** Adhesion of primary EOC cells on E-plates coated with 1% BSA (CTRL), 5 μg mL Vn, 5 μg mL Fn, 5 μg mL Lm or 15 μg mL Coll, with/without 10 nM RI-3, monitored in real time for 5 h by the xCELLigence system. Data represent mean ± SD from a quadruplicate experiment representative of 3 replicates. **b-c** Adhesion of EOC cells onto Vn in the absence or presence of 10 nM RI-3, monitored in real time for 3 h by the xCELLigence system. Data represent mean ± SD from a quadruplicate experiment representative of 3 replicates. Slopes represent the change rate of Cell Indexes generated in a 10–120 min time frame (**c**). **d** HPMCs were seeded in 24-well plates and allowed to attach for 20 h prior to seeding GFP-EOC cells suspended in complete medium plus diluents (none), or 10 nM RI-3 at 37 °C, 5% CO_2_. At the indicated times, cell associated fluorescence was assessed by a fluorescence plate reader. Data represent means ± SD of three independent experiments performed in triplicate with *P < 0.0001 vs none. **e-f** Mesothelium-invasion by primary EOC cells. HPMCs (5 × 10^3^ cells/well) were seeded in E-16-well plates in growth medium and allowed to adhere for 18 h to form a confluent monolayer. Then, EOC cells (2 × 10^4^ cells/well) were seeded in growth medium plus/minus 10 nM RI-3. Cells invasion was monitored in real-time as changes in Cell Index due to breaking of the monolayer integrity (**e**). Slopes represent the change rate of Cell Indexes generated in a 18–24 h time frame (**f**). Data represent mean ± SD from a quadruplicate experiment representative of 3 replicates

In order to assess whether the uPAR/FPR1 crosstalk dictates the capability of ovarian cancer cells to adhere onto Vn and mesothelium, a series of adhesion experiments was performed in the presence or the absence of 10 nM RI-3. Like SKOV-3 cells, primary EOC cells from patient #5 are able to adhere onto Vn, Fn, and Coll, although at a different extent (Fig. 6a) and the peptide RI-3 exclusively reduced cell adhesion onto Vn by 55% (Fig. 6a, b, c). When GFP-tagged EOC cells were seeded on a mesothelial cell monolayer in the presence/absence of 10 nM RI-3, and cell associated fluorescence was measured after 5, 10, 20, and 40 min, we found that, similarly to SKOV-3 cells, primary EOC cells adhere to mesothelium already after 5–10 min of incubation and that cell adhesion to mesothelium further increases over time. After 5, 10, 20, and 40 min, RI-3 caused a 72, 51, 47, and 46%, respectively, reduction of EOC cell adhesion onto mesothelium (Fig. 6d). Then, the ability of RI-3 to counteract mesothelial cell invasion by primary EOC cells was investigated as above described. An appreciable reduction of mesothelial cell monolayer integrity was achieved by EOC cells and RI-3, at 10 nM concentration, reduced by about 80% the capability of EOC cells to cross the mesothelial cell monolayer (Fig. 6e–f). Collectively, these findings demonstrate that RI-3 greatly reduces the uPAR84–95-induced and FPR1-mediated adhesion onto and invasion through mesothelium of primary EOC cells. They also pinpoint the possibility that FPR1 may be considered a new biomarker predicting the ability of EOC cells to adhere and subsequently invade mesothelium.

### FPR1 expression in human EOC tissues

**Fig. 7:**
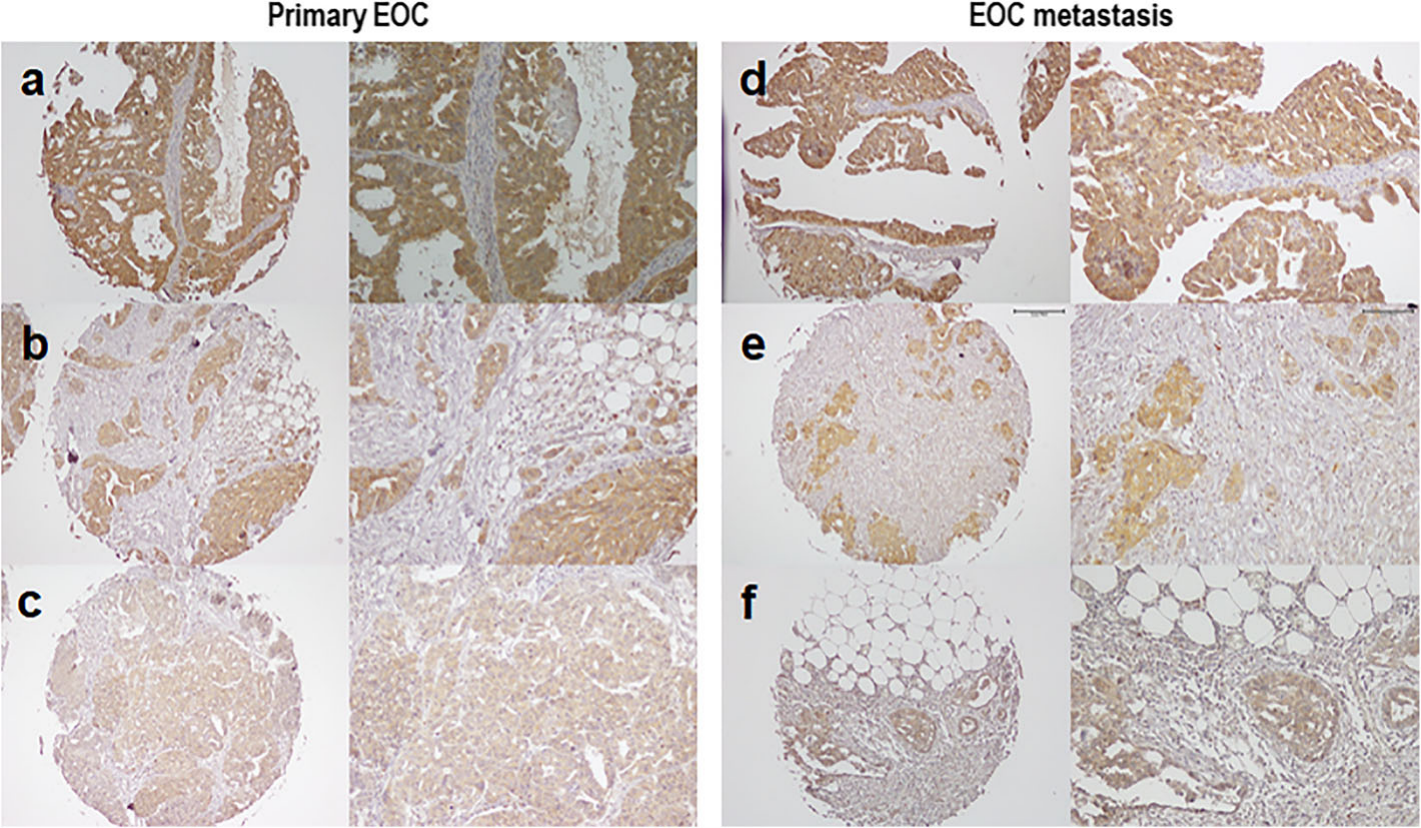
FPR1 expression in human EOC tissues. Ovarian cancer TMA slides were processed for IHC analysis of FPR1 expression. The three representative panels show intense (**a, d**), medium (**b, e**) and low (**c, f**) expression of FPR1 in primary (**a, b, c**) and metastatic (**d, e, f**) EOC tissues. Original magnifications: × 100 (left) and × 200 (right)

In light of the results presented above, we analyzed the FPR1 expression profile in EOC tissues using a TMA built with 37 EOC tissue samples. IHC highlighted variable expression of FPR1 both in the cytoplasm and on the plasma membrane of tumor cells (Fig. 7). FPR1 expression levels were evaluated by assessing either the percentages of positive tumor cells/TAM punch, either staining intensity of tumor cells, graded as faint (score 0), moderate (score 1), or intense (score 2). FPR1 scores were also correlated with the age, histotype, and FIGO stage. We found little FPR1 staining on plasma membranes and cytoplasm in 29.7% (*n* = 11) of cases in which the percentage of FPR1 positive cells ranges between 0 and 10%, with faint intensity. Conversely, the remaining 70.3% (*n* = 26) of cases exhibited moderate or intense FPR1 expression in at least 80% of tumor cells (Table 2). Although no statistical evaluation was applicable for the lack of representativeness of LGSC, OCCC and mEOC cases, we observed that FPR1 is mainly expressed in HGSC tumors, and that 80% positive tumor cells observed in 20 out of 28 HGSC tissues. Remarkably, a high percentage of moderate or intense FPR1 staining of EOC cells seems to correlate with FIGO stage III (Table 2) as FPR1 protein increased in a stepwise fashion from FIGO stage I (8%) to FIGO stage II (21.6%) to FIGO stage III (37.8%), suggesting a link between FPR1 abundancy to the malignant stages. No statistically significant correlation between the percentages of FPR1 positive cells in primary and matched metastatic tissues was found (Table 3). Interestingly, we observed that primary and matched metastases exhibit comparable expression levels of FPR1 with the exception of two cases in which FPR1 increases in metastatic lesions as compared to the primary ones and in a case in which metastatic cells are FPR1-negative (Table 3). Also, differences in the FPR1 expression levels between 3 HGSC primary lesions and matching metastases recovered after chemotherapy, during the second-look laparotomy, were not significant. Although preliminary, these experiments detected for the first time FPR1 in human EOC tissues, suggesting FPR1 as a potential biomarker of aggressive EOC. Finally, the notion that uPAR overexpression associates with poor prognosis and unfavorable clinical outcome of EOC patients [17], together with the finding that most malignant EOC tissues express high levels of FPR1, may constitute the rationale for new therapeutic strategies that include inhibitors of the uPAR84–95/FPR1 interaction.

**Table 2:** Relationship between FPR1 expression and clinicopathological features of ovarian cancer tissues

			FPR1 staining (%)			FPR1 staining (intensity)		
			Neg ≤ 10	Pos ≥ 80	*P-*value^a^	0	1	2
EOC tissues	n.cases	37 (100.0)	11 (29.7)	26 (70.3)	0.391	11 (29.7)	17 (45.9)	9 (24.3)
Age	**≤**50	8 (21.6)	1 (2.7)	7 (18.9)	0.08	1 (2.7)	3 (10.8)	4 (10.8)
	> 50	29 (78.4)	10 (27.0)	19 (51.4)	0.11	10 (27.0)	14 (37.8)	5 (13.5)
Histotype	LGSC	3 (8.1)	1 (2.7)	2 (5.4)	0.612	1 (2.7)	2 (5.4)	0 (0.0)
	HGSC	28 (75.7)	8 (21.6)	20 (54.1)		8 (21.6)	13 (35.1)	7 (18.9)
	OCCC	5 (13.5)	1 (2.7)	4 (10.8)		1 (2.7)	2 (5.4)	2 (5.4)
	mEOC	1 (2.7)	1 (2.7)	0 (0.0)		1 (2.7)	0 (0.0)	0 (0.0)
FIGO	I	6 (16.2)	3 (8.1)	3 (8.1)	0.42	3 (8.1)	2 (5.4)	1 (2.7)
	II	9 (24.3)	1 (2.7)	8 (21.6)		1 (2.7)	4 (10.8)	4 (10.8)
	III	21 (56.8)	7 (18.9)	14 (37.8)		7 (18.9)	10 (27.0)	4 (10.8)
	IV	1 (2.7)	0 (0.0)	1 (2.7)		0 (0.0)	1 (2.7)	0 (0.0)

^a^*P* - value calculated using Pearson’s Chi-square (χ^2^) test

**Table 3:** FPR1 expression in primary and metastatic ovarian cancer tissues

HISTOTYPE	FIGO	P^a^		M^b^	
		≥ 80% staining	intensity	≥ 80% staining	intensity
LGSC	II	pos	1	pos	2
LGSC	III	pos	1	pos	1
HGSC	III	pos	1	pos	1
HGSC	III	pos	1	pos	1
HGSC	III	pos	1	pos	1
HGSC	IV	pos	1	pos	1
HGSC	III	pos	1	pos	1
HGSC	II	pos	2	pos	1
HGSC	III	pos	2	pos	2
HGSC	II	pos	2	pos	1
HGSC	III	neg	0	pos	1
HGSC	III	neg	0	pos	1
HGSC	III	pos	1	neg	0
HGSC	III	neg	0	neg	0
OCCC	II	pos	2	pos	2
OCCC	III	pos	1	pos	1

^a^Primary EOC

^b^Intra-abdominal EOC metastases

## Discussion

Epithelial Ovarian carcinoma is deadliest gynecological malignancy, as the majority of patients with EOC are first diagnosed when peritoneal metastases have already formed. EOC cells detach from the primary cancer as single or clusters of tumor cells, carried by ascitic fluid and attach to mesothelium lining the peritoneal cavity, establishing metastasis [12, 13, 46]. Development of experimental models to study the mechanism of ovarian cancer metastasis is challenging, as conventional approaches used to study tumor diffusion do not apply to the peculiar EOC trans-coelomic dissemination. Herein, a novel technology that records in real-time impedance changes proportional to the number of invading cells was used to analyze mesothelial invasion. This model may allow to monitor the early events in peritoneal anchoring and invasion of tumor cells and could represent a suitable model to test potential anti-invasive therapeutics.

In this study, we provide evidence that EOC adhesion to the mesothelium and subsequent spreading could be blocked by the recently identified RI-3 peptide interfering with the interaction of uPAR with FPR1, both regulators of cell adhesion and invasion [36, 38]. In agreement with the notion that vitronectin is the main mesothelium-associated ligand [46–50], SKOV-3 ovarian cancer and primary EOC cells, the last derived from a human HGSC tissue sample, markedly adhere to Vn matrices as well as to a re-constructed mesothelial layer. Under these conditions, cell adhesion onto Vn is uPAR84–95-dependent and FPR1-mediated for the following reasons; i) cell adhesion to Vn decreases to the basal level in the presence of anti-uPAR84–95 Abs, but not by R3 anti-uPAR mAb which does not recognizes the uPAR84–95 sequence, being directed to the D1 uPAR domain; ìì) cells fail to adhere onto Vn when desensitized with an excess of fMLF or SRSRY that allow FPR1 to internalize thereby making it unavailable on the cell membrane [38]; ììì) RI-3 peptide blocking FPR1 anchored on cell membrane and unable to signal [38], abrogates EOC cell adhesion onto Vn; ìv) human EOC cells lacking of both uPAR and FPR1 poorly adhere onto Vn.

**Fig. 8:**
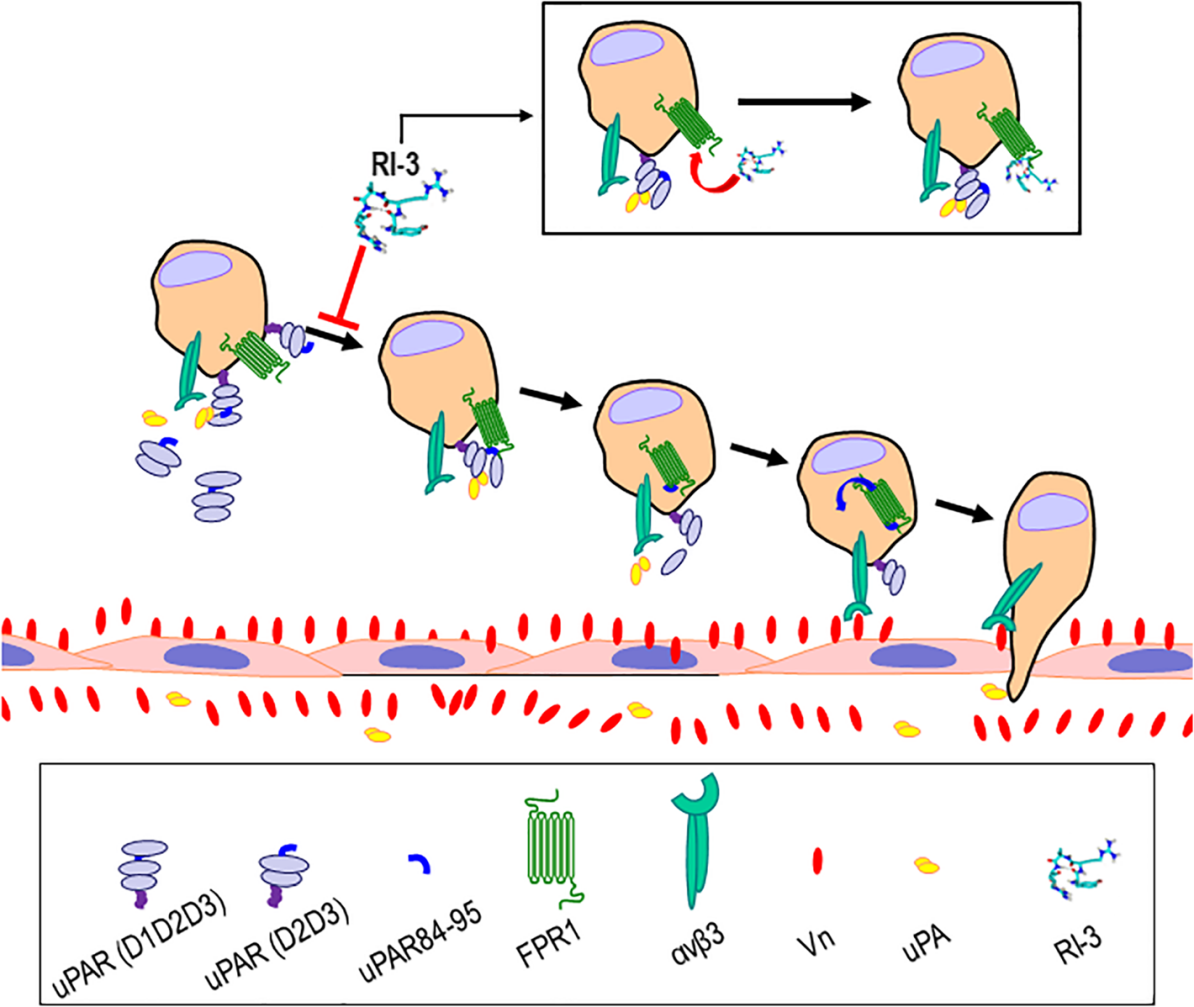
Schematic diagram of proposed mechanism of RI-3 inhibitory effects on EOC cell adhesion. After detachment from the primary tumor, EOC cells float in the peritoneal fluid. EOC cells able to adhere to mesothelial ECM components via integrins may penetrate mesothelial cell layers. Intact uPAR or uPAR-derived fragments containing the 84–95 sequence that are expressed on EOC cell surface or may be released in the ascitic fluid, bind to FPR1 generating a cascade of events. Once engaged, FPR1 internalizes, triggering αvβ3 vitronectin receptor activation which, in turn, allows EOC cells to adhere onto mesothelial Vn. Subsequently, proteases within the mesothelial milieu allow EOC cells to invade the mesothelial cell layer where they proliferate and form metastases. This process may be counteracted by nanomolar concentrations of RI-3 peptide, blocking the assembly of uPAR/FPR1 complexes

Among the signaling pathways active to control metastatic spread of cancers are the members of activator protein-1 (AP-1) family transcription factors, including c-Fos [51]. This transcriptional activator binds to AP-1 motifs localized also in the uPA and uPAR promoter regions, upregulating their mRNA synthesis [14].. When expressed on cell surface, uPAR associates with various integrins in large molecular complexes [52], and regulates integrin function directly, by interacting with the alpha chain of integrin, possibly, through “lateral” interactions [53, 54], or indirectly, through its uPAR 84–95 sequence that has been shown to trigger integrin αvβ3 activation with an inside-out mechanism, upon binding to FPR1 [26]. In 2012, we reported that the substitution of the Ser90 in the uPAR84–95 sequence generates a dominant-negative variant of uPAR that prevents agonist-triggered FPR1 activation and internalization, decreases binding and adhesion to Vn, and inhibits uPAR/vitronectin receptor association [55]. As a working model for RI-3-dependent inhibition of EOC cell adhesion onto Vn, we propose that the peptide blocks uPAR-triggered FPR1 activation, which ultimately impairs αvβ3 activation (Fig. 8). Since alpha-v integrin receptor is known to be involved in ovarian cell adhesion to mesothelium [56] it will be interesting to investigate whether αvβ3 integrin inhibitors may synergize with RI-3 inhibitor in reducing mesothelial invasion of EOC cells. Furthermore, since uPA/uPAR complexes regulate entering of cancer stem cells a dormant state when unable to establish integrin-mediated interactions, we plan to investigate whether FPR1 expression/function is involved in this process [57].

To the best of our knowledge, no data are available in literature on the FPR1 expression in EOC cells. Here, we show for the first time a diffuse moderate/intense staining of FPR1 in the 70% of examined EOC tissue samples, showing high-level expression of this receptor in most tissue samples. In this respect, we plan to extend the number of cases to evaluate the power of FPR1 as a negative prognostic marker, as well as the possible correlation between FPR1 expression levels, histotype, and FIGO staging. For EOC patients, the risk of relapse is high, and most of them develop platinum-resistant progressive disease, which restricts available therapeutic options. Emerging data suggest that the cell-signaling activity of uPAR may allow cancer cells to “escape” from the cytotoxic effects of targeted anticancer drugs [58]. It will be interesting to analyze whether the acquired resistance to chemotherapy of EOC patients correlates with changes in the uPAR and/or FPR1 expression levels. Intriguingly, in a single HGSC case, FPR1 staining of ciliated epithelium from fallopian tubes adjacent to neoplastic areas was observed. Because it has been reported that fallopian tube epithelium gives rise to serous ovarian cancers [59–61], we are planning to analyze in-depth the expression levels of FPR1 in areas adjacent to the EOC tissues.

To date, most therapeutics strategies targeting uPAR do not shown robust anti-tumor activity [62, 63]. On the other hand, despite a variety of natural and synthetic compounds that interfere with FPR1-dependent pathways antagonizing FPR1functions have been described [64, 65], the majority had off-target effects, discouraging their clinical development. In this contest, the selective impairment of uPAR-mediated FPR1 triggered signaling by RI-3 is expected does not affect other functions regulated by FPR1. Indeed RI-3 does not affect cell proliferation and was well tolerated in vivo when administered to mice with no visible side-effects and no change of body weight versus vehicle-treated animals [36]. Now we found that RI-3 not only prevents EOC cell adhesion to mesothelium but also dramatically decreases the ability of EOC cells to cross mesothelial cell monolayers. Although we not yet studied the efficacy of RI-3 in preventing EOC attachment to mesothelium and subsequent invasion in animal models, our findings suggest that RI-3 may be considered as a valid prototype for the development of new therapies which, combined with conventional chemotherapy, should counteract intra-abdominal dissemination of EOC cells.

## Conclusions

Collectively, our findings identify FPR1 as novel, valuable, marker for predicting the propensity of EOC cells to adhere and subsequently invade mesothelium. Furthermore, they suggest FPR1 as a new therapeutic target to be blocked by inhibitors of the uPAR84–95/FPR1 crosstalk for the treatment of metastatic ovarian cancer.

## Supplementary information

**Additional file 1: Figure S1.** Adhesion and mesothelial invasion properties of uPAR and FPR1 lacking EOC cells. **a.** Fax analysis of human ovarian carcinoma A2780 cells with (CD87)-APC-conjugated anti-uPAR or PE-conjugated FPR1 antibodies. **b.** Cell adhesion of A2780 cells on 1% BSA (CTRL), Vn-, Fn-Lm or Coll-coated plates, in the presence or the absence of 10 nM RI-3. The number of adherent cells was expressed as a percentage of CTRL. Data are the means ± SD of three independent experiment. **c.** Mesothelium invasion by A2780 cells. HPMCs (5 × 10^3^ cells/well) were seeded in E-16-well plates and allow to adhere for 20 h until they form a confluent monolayer. Then, A2780 cells suspended in growth medium plus/minus 10 nM RI-3 were seeded onto the mesothelial cell monolayer and invasion of mesothelium by A2780 cells was monitored in real-time as changes in Cell Index due to breaking of the monolayer integrity. Data represent mean ± SD from a quadruplicate experiment representative of 2replicates. **Figure S2.** Uncropped images of immunoblots from Fig. 5c.

## Data Availability

All data generated or analyzed during this study are included in this published article and its supplementary information file. Further details are available from the corresponding author on reasonable request.

## Abbreviations

ECM: Extracellular matrix
EOC: Epithelial ovarian cancer
FBS: Foetal bovine serum
fMLF: N-formyl-Met-Leu-Phe
FPR1: Formyl-peptide receptor type 1
HGSC: High-grade serous carcinoma
HPMC: Human peritoneal mesothelial cell
IHC: Immunohistochemistry
LGSC: Low-grade serous carcinoma
mEOC: Mucinous ovarian carcinoma
NRS: Normal rabbit serum
OCCC: Clear cell carcinoma
PBS: Phosphate-buffered saline
TBS: Tris buffered saline
TMA: Tissue microarray
uPA: Urokinase Plasminogen Activator
uPAR: Urokinase Receptor
