# Supplementary material for: Targeting the Formyl Peptide Receptor type 1 to prevent the adhesion of ovarian cancer cells onto mesothelium and subsequent invasion
Source: J Exp Clin Cancer Res. 2019 Nov 8;38:459. doi: 10.1186/s13046-019-1465-8 (PMC6839174; doi:10.1186/s13046-019-1465-8)

Supplementary Figure S1. Adhesion and mesothelial invasion properties of uPAR and FPR1 lacking EOC cells

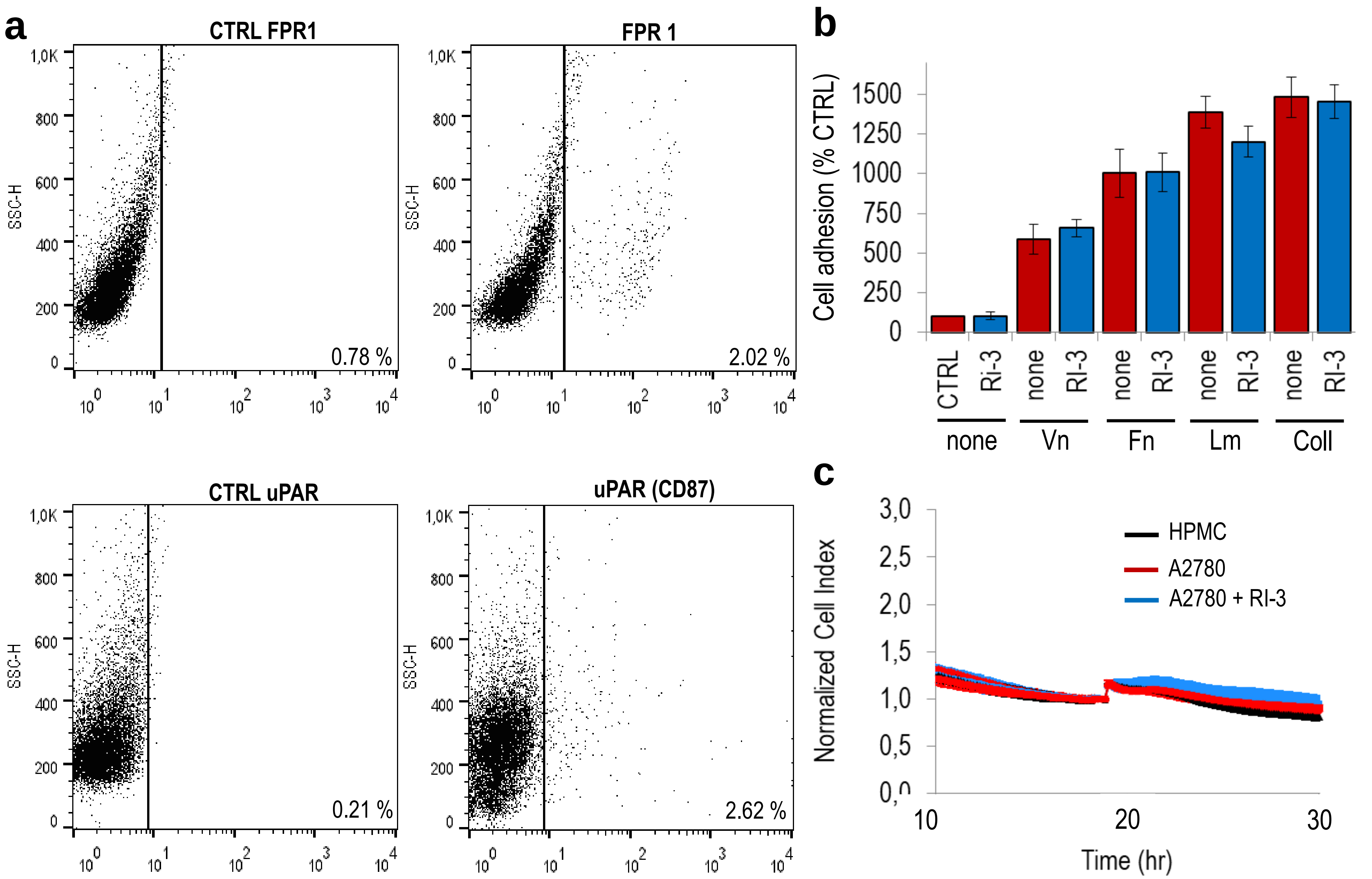

Supplement: Supplementary file 1 — Additional file 1: Figure S1. Adhesion and mesothelial invasion properties of uPAR and FPR1 lacking EOC cells. a. Fax analysis of human ovarian carcinoma A2780 cells with (CD87)-APC-conjugated anti-uPAR or PE-conjugated FPR1 antibodies. b. Cell adhesion of A2780 cells on 1% BSA (CTRL), Vn-, Fn-Lm or Coll-coated plates, in the presence or the absence of 10 nM RI-3. The number of adherent cells was expressed as a percentage of CTRL. Data are the means ± SD of three independent experiment. c. Mesothelium invasion by A2780 cells. HPMCs (5 × 10^3^ cells/well) were seeded in E-16-well plates and allow to adhere for 20 h until they form a confluent monolayer. Then, A2780 cells suspended in growth medium plus/minus 10 nM RI-3 were seeded onto the mesothelial cell monolayer and invasion of mesothelium by A2780 cells was monitored in real-time as changes in Cell Index due to breaking of the monolayer integrity. Data represent mean ± SD from a quadruplicate experiment representative of 2replicates. Figure S2. Uncropped images of immunoblots from Fig. 5c. [file 13046_2019_1465_MOESM1_ESM.zip › Supplementary Figure 1.pdf]
